# Substituted flavones: a promising scaffold in the fight against malaria

**DOI:** 10.1186/1475-2875-13-S1-P64

**Published:** 2014-09-22

**Authors:** Flore Nardella, Valérie Collot, Sylvia Stiebing, Marcel Kaiser, Martine Scmitt, Ermanno Candolfi, Catherine Vonthron-Sénécheau

**Affiliations:** 1 Laboratoire d’Innovation Thérapeutique, Faculté de Pharmacie, UMR CNRS-Unistra 7200, Illkirch, France; 2 Faculté de Médecine, Institut de Parasitologie et de Pathologie Tropicale de Strasbourg, Strasbourg, France; 3 Centre d’Etudes et de Recherches sur le Médicament en Normandie, Université de Caen Basse-Normandie, Caen, France; 4 Swiss Tropical and Public Health Institute, Basel, Switzerland

## Background

Ever since 2008, when evidence of artemisinin resistant malaria was highlighted in Western Cambodia [1], the need for new drugs with an original structure and novel mechanisms of action is even more pressing. The study of traditional remedies such as Cinchona bark or Artemisia aerial parts led to the discovery of the most potent antimalarials, bearing out that nature is still an incredible source of inspiration. Based on this approach, we are developing new synthetic antimalarial agents with an original structure inspired by nature.

## Material and methods

*In vitro* antiplasmodial activity is evaluated against two strains of *P. falciparum* (multiresistant K1 and chloroquino-resistant 7G8) with two different methods: the inhibition of ^3^[H]-hypoxanthine incorporation assay and the Plasmodium-LDH immunoassay detection kit. Cytotoxicity assays are performed on two murine cell types (L6 and Hepa) in order to estimate the selectivity index, with two different viability tests: the resazurine assay and an MTT-based approach. *In vivo* activity is performed according to Peters’ experiment [2] on *P. berghei* ANKA murine model at a dosing regimen of 100 mg/kg intraperitoneally.

## Results

The isolation of an *in vitro* active biflavonoid from Campno-sperma panamense (Anacardiaceae, IC_50_ = 480 nM, *P. falciparum* K1), led us to the development of simplified synthetic analogs (MR series) with improved pharmacological and pharmacokinetic profiles. Notably one of them (MR70) exhibits a partial *in vivo* antimalarial activity with a reduction of parasitaemia by 45% on day 4.

## Conclusion

We described here the first substituted flavone showing an *in vivo* antimalarial activity in a murine model. This scaffold should be promising in the fight against malaria. To understand its mechanism of action, we are currently running differential metabolomics analysis by solid NMR (coll. Pr. J. I. Namer, Strasbourg, France) to highlight some potential impact on Plasmodium metabolic pathways.
